# Supplementary material for: Genome-Wide Identification and Characterization of Oil-Body-Membrane Proteins in Polyploid Crop Brassica napus
Source: Plants (Basel). 2022 Aug 29;11(17):2241. doi: 10.3390/plants11172241 (PMC9460193; doi:10.3390/plants11172241)
Supplement: Supplementary file 1 [file plants-11-02241-s001.zip › Supplementary Files/Supplementary Figures.pdf]

Supplementary Figures

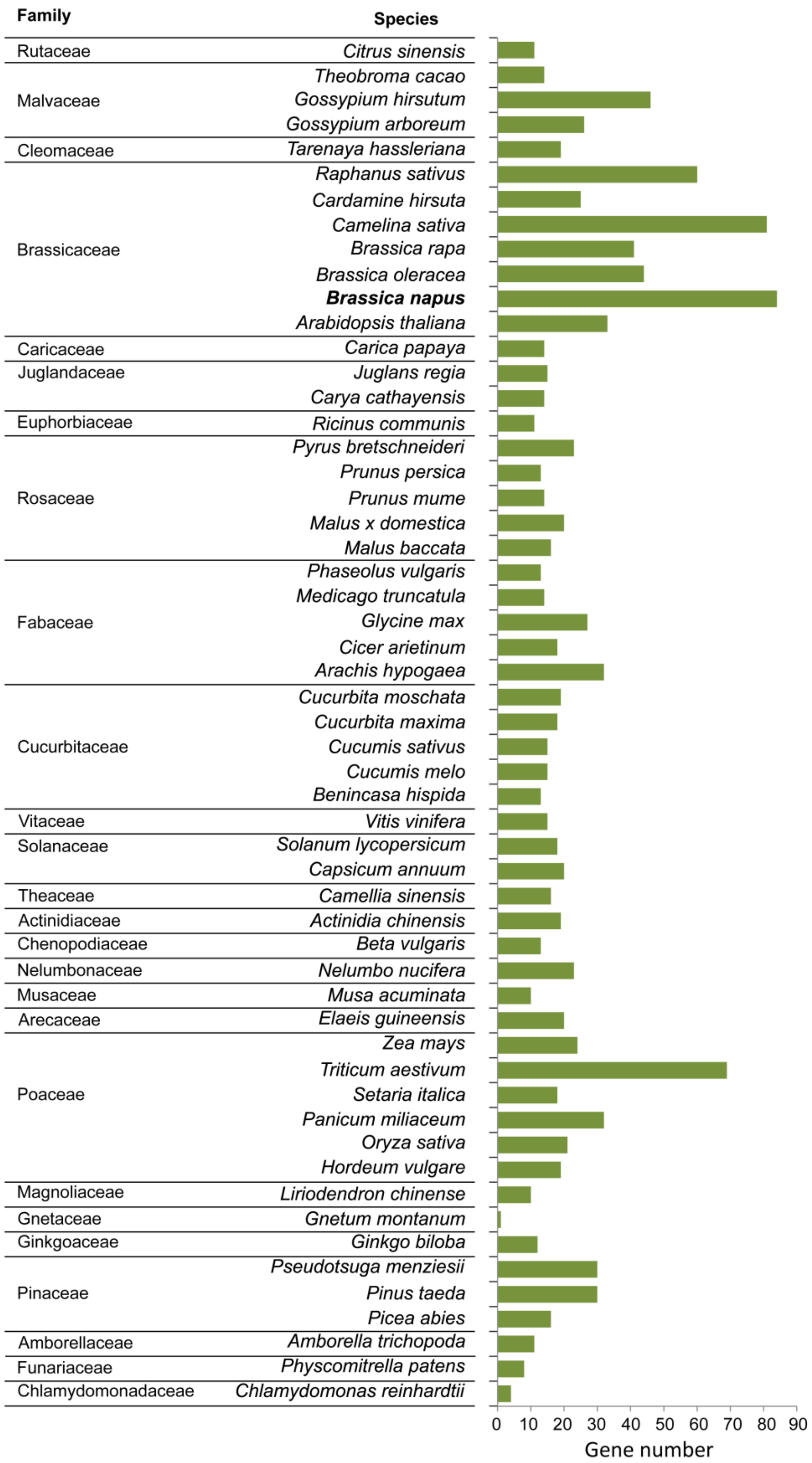

Figure S1. Number of *OBMP* genes identified from each of the plant genomes. Family and species are listed at the left. The *Brassica napus* is marked by bold font.

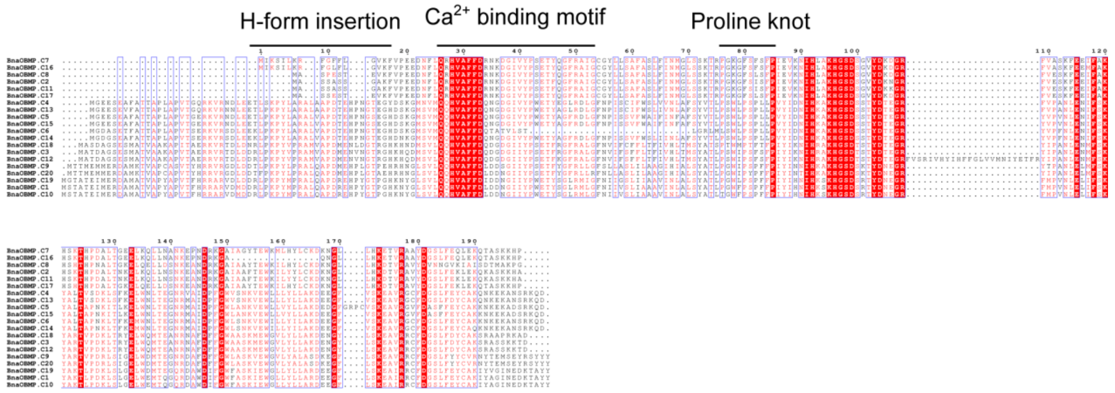

Figure S2. Pileup of the sequences of all caleosins in *B. napus* aligned with the MUSCLE method in MEGA software. The H-form insertion, Ca^{2+} binding motif, and proline knot are indicated.

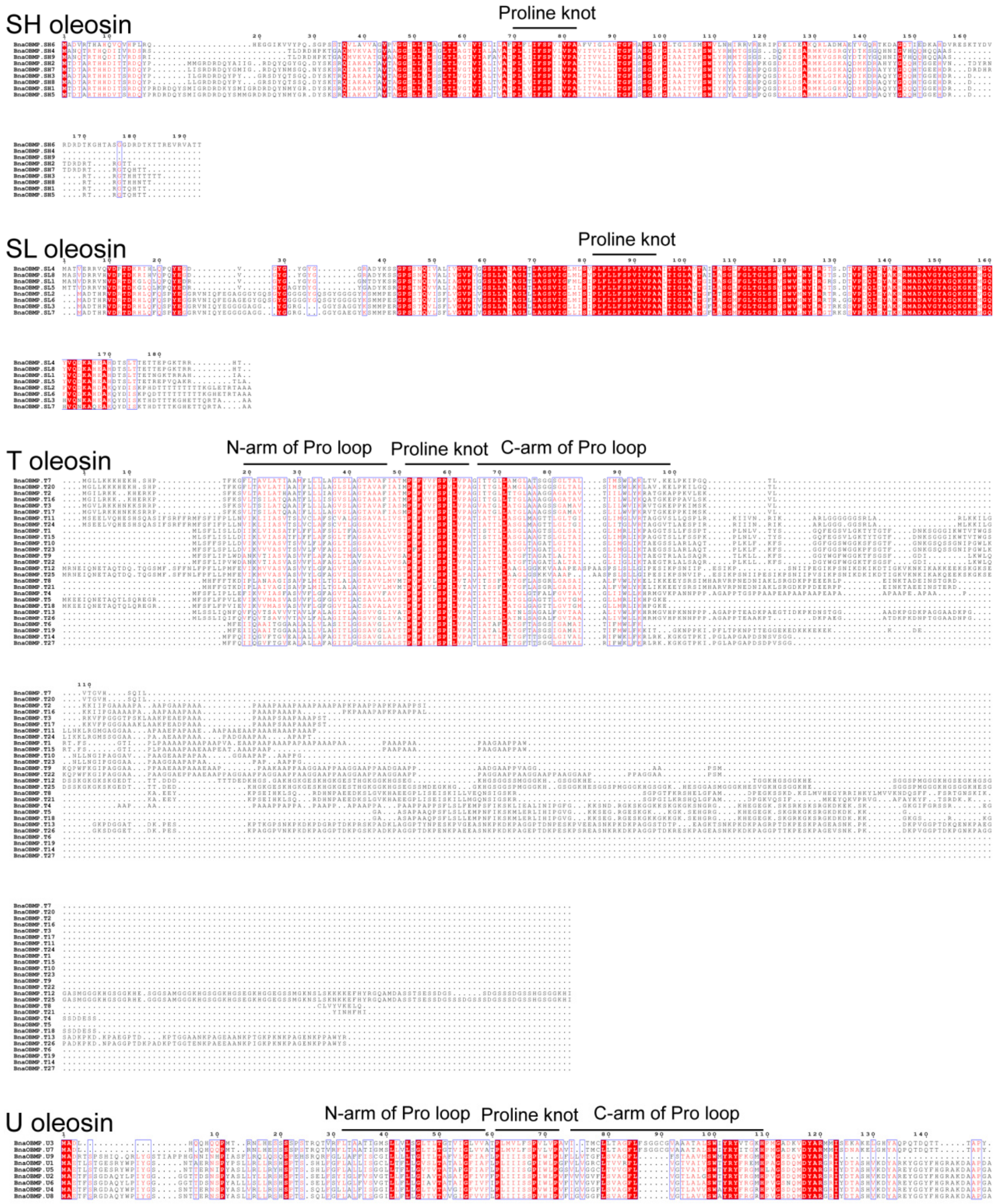

Figure S3. Pileup of the sequences of all U, SL, SH and T oleosins in *B. napus* aligned with the MUSCLE method in MEGA software. The proline knot of SH oleosins, proline knot of SL oleosins, and N-arm of Pro loop, proline knot, and C-arm of Pro loop of T oleosins and U oleosins are indicated.

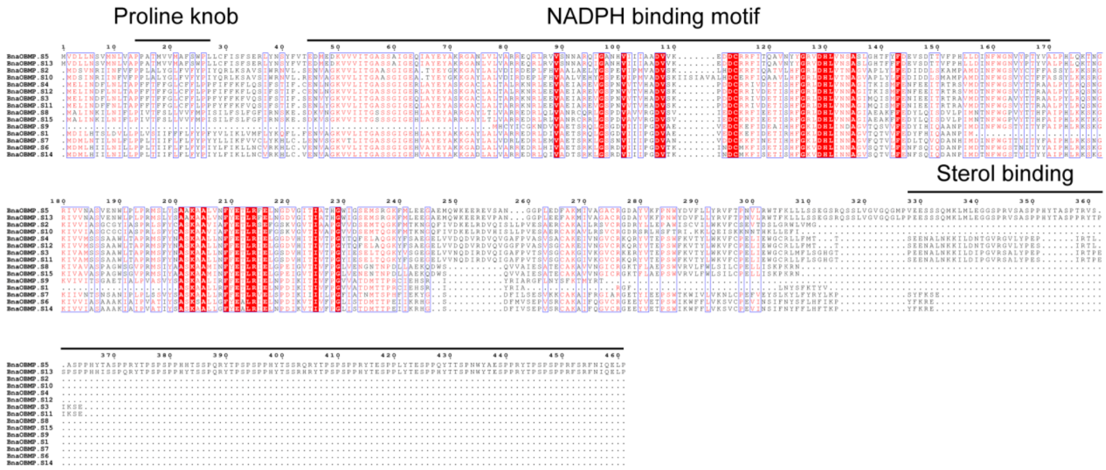

Figure S4. Pileup of the sequences of all steroleosins in *B. napus* aligned with the MUSCLE method in MEGA software. The proline knob, NADPH binding motif, and sterol binding domain are indicated.

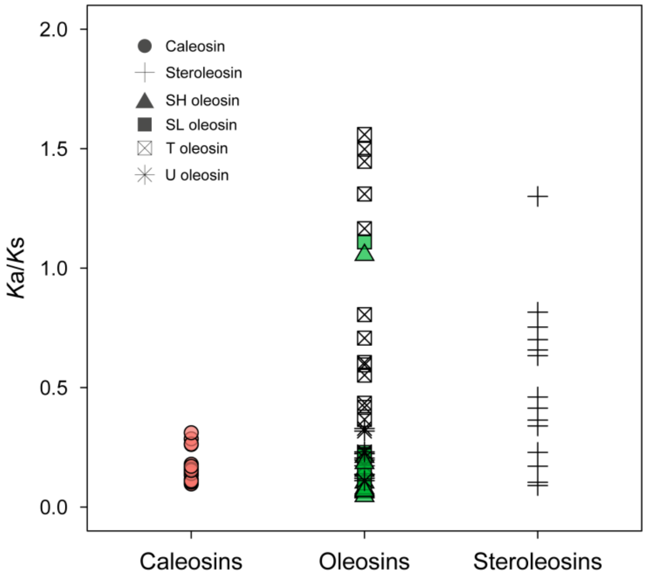

Figure S5. Selective pressures for OBMP genes in *B. napus* as revealed in non-synonymous and synonymous substitution (*Ka*/*Ks*) ratios. The *Ka*/*Ks* ratio for each oleosin, caleosin and steroleosin in *B. napus* relative to its ortholog in *A. thaliana* was calculated with KaKs_Calculator and shown in different shapes representing the different OBMP lineages.

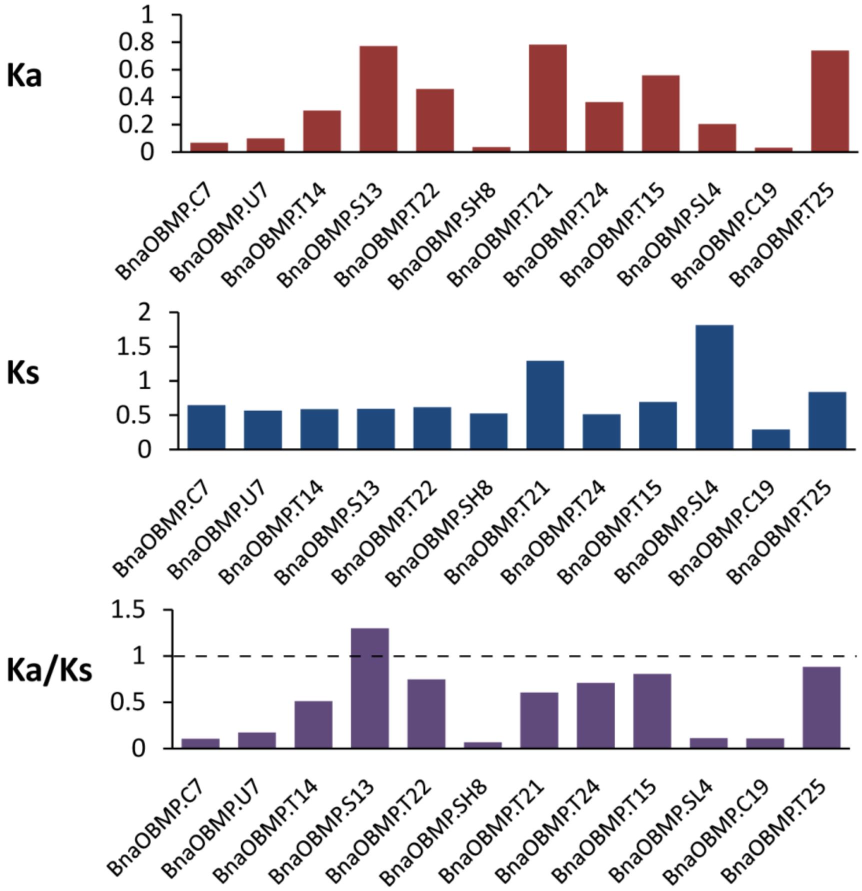

Figure S6. *Ka, Ks*, and *Ka*/*Ks* ratios of 12 OBMP genes under selection in *B. napus*. The *Ka, Ks*, *Ka*/*Ks* ratio values for each *OBMP* genes in *B. napus* relative to its ortholog in *A. thaliana* was calculated with KaKs_Calculator. The dash line indicated the *Ka*/*Ks* ratio equal to 1.

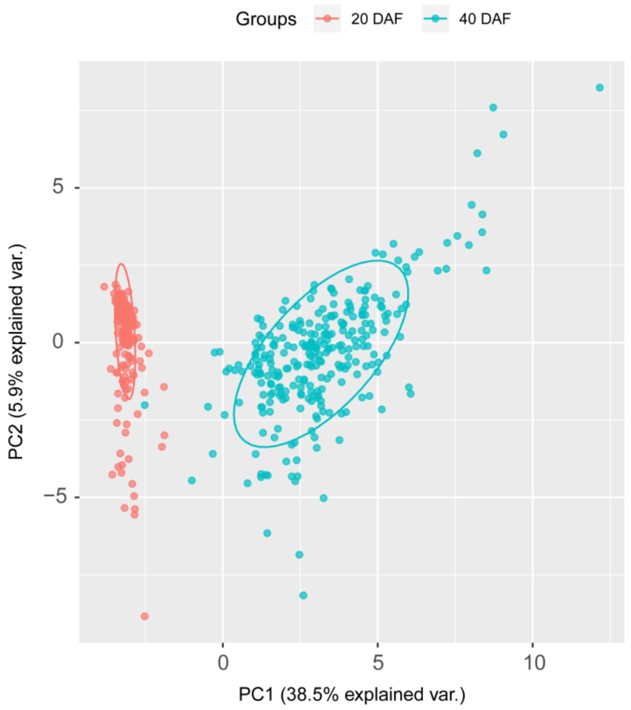

Figure S7. PCA plot of all the seed samples of 280 *B. napus* accessions. Each dot represents a sample, which is labeled by group. Two major sample clusters are indicated: 20 DAF (red) and 40 DAF (cyan). The first two principal components explained about 44.5 % of the total variation.

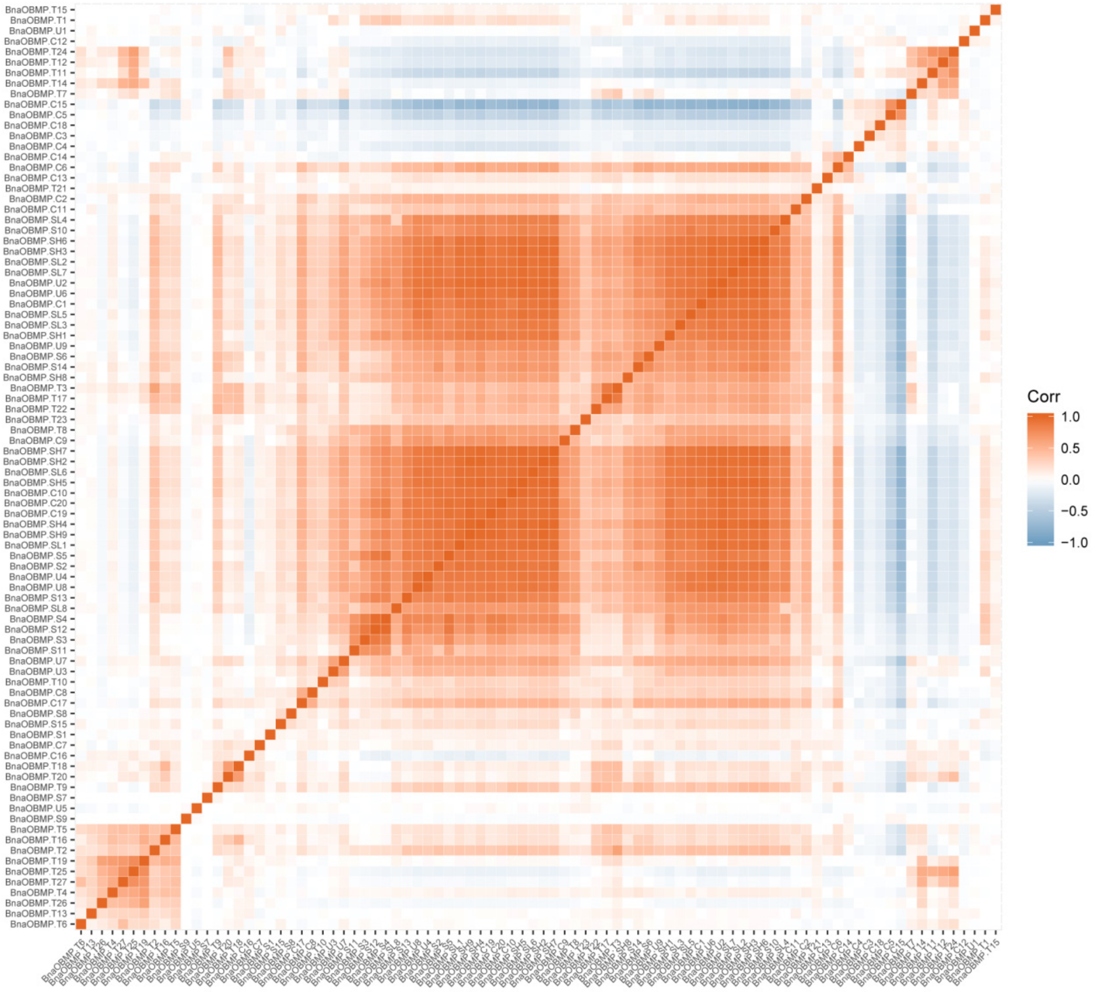

Figure S8. Correlation matrix of gene expression levels among *BnaOBMP* genes. Pearson correlation coefficients (PCCs) and correlation *p*-values were calculated based on the seed RNA-seq data of 280 *B. napus* accessions at 20 DAF and 40 DAF. The color of each cell indicate the PCC value between *BnaOBMP* gene pair from negative (blue) to positive (orange).

Supplementary Tables

Table S1. Summary information of *OBMP* gene family in *A. thaliana*, *B.rapa* and *B.oleracea.*

Please see the Excel table.

Table S2. Statistic of the variations of *BnaOBMP* genes.

Please see the Excel table.

Table S3. Hub genes of *BnaOBMP*-GRN network in *B. napus.*

Please see the Excel table.
